# Development and Application of Poly (Lactic Acid)/Poly (Butylene Adipate-Co-Terephthalate)/Thermoplastic Starch Film Containing Salicylic Acid for Banana Preservation

**DOI:** 10.3390/foods12183397

**Published:** 2023-09-11

**Authors:** Jian Ding, Yi Hao, Boqiang Liu, Yunxia Chen, Li Li

**Affiliations:** 1 College of Food Science and Technology, Shanghai Ocean University, Shanghai 201306, China; jian-d-shou@foxmail.com (J.D.); luosiliners@163.com (Y.H.); 15872773547@163.com (B.L.); 2 School of Mechanical Engineering, Shanghai Dianji University, Shanghai 201306, China

**Keywords:** salicylic acid, banana, oxygen transmission rate, packaging film, preservation

## Abstract

Bananas are susceptible to the effects of endogenous enzymatic, leading to their rapid decay and deterioration. In order to mitigate economic losses and prolong the shelf life of bananas, the objective of this study was to develop a new and green gas-regulating packaging film. In this study, an active gas-regulating packaging film was prepared by extrusion, with mobil composition of matter (MCM)-41 loaded with salicylic acid (SA) as the active agent and poly (lactic acid) (PLA), poly (butylene adipate-co-terephthalate) (PBAT), and thermoplastic starch (TPS) as the base materials. The obtained films included PLA/PBAT/TPS, PLA/PBAT/TPS-SA, and PLA/PBAT/TPS-MCSA. These films were subsequently applied to banana preservation. The study focused on the variations in soluble solid content (SSC), rate of weight loss (RWL), malondialdehyde (MDA) content, and polyphenol oxidase (PPO) activity of bananas during the preservation process. The results showed that, compared with the PLA/PBAT/TPS film, the oxygen transmission rate of the PLA/PBAT/TPS-MCSA film increased from 384.36 ± 22.06 cm^3^·m^−2^·24 h^−1^·0.1 MPa^−1^ to 543.10 ± 3.47 cm^3^·m^−2^·24 h^−1^·0.1 MPa^−1^. Throughout the preservation period, the PLA/PBAT/TPS-MCSA film exhibited superior performance, effectively retarding the increase in banana SSC, RWL, and MDA while inhibiting the elevation of PPO activity and prolonging the shelf life of bananas by 4–5 days. However, this study needs to further investigate the mechanism of function of MCM-41 loaded with SA in banana preservation.

## 1. Introduction

Banana is a typical tropical and subtropical fruit with sweet and tasty flesh that is rich in nutrients [1]. But in the storage process of bananas, which generally can ripen naturally in 3–7 days, softening of the flesh, browning of the peel and the core, and reduction of the nutrient content occur, which greatly affect the quality and commercial value of the fruit [2]. Physical or chemical treatments such as post-harvest low-temperature storage, spraying, low-oxygen packaging, and 1-Methylcyclopropene (1-MCP) are currently the most common preservation methods for bananas [3]. However, low-temperature storage will lead to cold damage to bananas [4], and a single spray treatment is not suitable for long-term storage and long-distance transportation of bananas [5]. Therefore, whether from the economic point of view or from the storage and transportation point of view, seeking a preservation technology that can improve the post-harvest quality and extend the storage life of bananas has an important economic value.

Salicylic acid (SA) is a *β*-hydroxy phenolic acid that serves as a natural and safe plant growth regulator, participating in the physiological and metabolic processes of plant growth, development, and stress response [6,7]. Exogenous SA has been recognized for its ability to inhibit microbial growth and reproduction, thereby retarding the decay of bananas. Its penetrating ability through cell walls contributes to its antimicrobial properties, positioning SA as a natural, efficient, non-toxic, and cost-effective plant growth regulator widely adopted in the food preservation industry [7]. Furthermore, SA has been demonstrated the capacity to mitigate quality loss and hardness decline during the storage of strawberries and grapes, preserving their sensory attributes and physiological parameters. Notably, SA has been shown to inhibit the internal browning of peaches under refrigeration conditions [8,9,10]. Charles et al. [11] reported that SA exerts its anti-ethylene effects through multiple physiological and metabolic regulations, including the reduction of 1-aminocyclopropane-1-carboxylic acid (ACC) oxidase synthesis and activity, thus impeding the conversion of ACC to ethylene. Moreover, SA delays fruit softening by inhibiting cell wall-degrading enzyme activity [12,13].

However, the incorporation of SA into food packaging films often encounters challenges such as uneven dispersion and controlled release. Addressing these issues effectively can significantly enhance the applicability of SA in the realm of food industry preservation films. Mobil composition of matter (MCM) represents a class of molecular sieves distinguished by their ordered mesoporous or macroporous structures. Among these, MCM-41 stands out due to its robust thermal and hydrolytic stability, significant specific surface area, and pore size [14,15]. Vilaca et al. [16] reported a comparison was made between various mesoporous materials, including MCM-41, SBA-15, and microporous zeolite, as well as other carriers. The study revealed that MCM-41 loaded with SA exhibited superior performance and the most effective slow-release effect, potentially providing a solution to the challenge of poor dispersion and the difficult, slow release of SA.

In this study, an active packaging film with gas-regulating properties was developed using poly (lactic acid) (PLA), poly (ethylene glycolic acid)-butylene terephthalate (PBAT), and thermoplastic starch (TPS) as the matrix and MCM-41 loaded with SA as the active component. Zhao et al. [17] successfully developed an active packaging film that can regulate the relative humidity inside the packages and inhibit the autolysis of straw mushrooms using PLA/PBAT/TPS, so we would like to utilize this matrix containing salicylic acid to regulate the gas composition inside the packages. The application of this active packaging film was explored for banana preservation. The impact of the active packaging on banana quality was assessed through measurements of O_2_ and CO_2_ levels, soluble solid content (SSC), rate of weight loss (RWL), malondialdehyde (MDA) content, polyphenol oxidase (PPO) activity changes, and sensory evaluation of packaged bananas. The primary objective of this research was to devise an active packaging film with gas-regulating capabilities and to evaluate its influence on banana storage quality. The outcomes of this study hold the potential to serve as a reference for the development of active packaging with gas-regulating properties. 

## 2. Materials and Methods

### 2.1. Materials

PLA 721 was purchased from Hisun Biomaterials Co., Ltd. (Taizhou, China) and had a mean molecular weight (Mw) of 210 kDa and polydispersity of 1.72. PBAT C1200 was obtained from Wantong Chemical Co., Ltd. (Zhuhai, China). SA was purchased from Sinopharm Group Co., Ltd. (Shanghai, China). MCM-41 (silicon-to-aluminum ratio 25:30) was purchased from Beike New Material Technology Co., Ltd. (Beijing, China). All chemicals were analytical grade and used as received from Macklin Biochemical Technology Co., Ltd. (Shanghai, China). The bananas were supplied from the fresh fruit and vegetable market in Pudong Shanghai, China.

### 2.2. Preparation of MCM-41 Loaded with SA Powder

The MCM-41 loaded with SA (MCSA) composite powder was prepared according to the previous method [18]. Briefly, MCM-41 was subjected to an overnight drying process in a vacuum oven at 90 °C to ensure the complete removal of moisture from its pores and to prevent residual moisture from adversely affecting the subsequent loading of SA. A precise quantity of 300 mL of acetone was then added to the beaker, followed by the introduction of 7.92 g of SA powder into the solution. The mixture was stirred at room temperature until complete dissolution was achieved. Subsequently, 20 g of MCM-41 was gradually introduced into the aforementioned solution in several installments. The resulting mixture was stirred magnetically at 30 rpm for 60 h under ambient conditions to attain the ultimate suspension. Finally, the mixture underwent multiple filtration steps to obtain MCSA crystals. The resulting MCSA crystals were subjected to vacuum drying at 90 °C for 12 h, followed by gentle pulverization using a mortar, resulting in the production of white complex MCSA powder. This powder was subsequently hermetically sealed within aluminum foil bags for storage.

### 2.3. Preparation of Film

Referring to the approach of Zhao et al. [17,19], glycerol and corn starch were compounded in a certain proportion to prepare TPS. Subsequently, 2 wt% SA and 2 wt% MCSA powders were separately melt-blended with PLA, PBAT, and TPS, and the material composition of the specific film preparation is shown in Table 1. Modified pellets were obtained through melt extrusion using a twin-screw extruder (LSSHJ-20, Shanghai, China), followed by film extrusion using a single-screw extruder (LSJ-20, Shanghai, China). The film thickness was controlled at around 35 ± 3 μm. Three kinds of films were marked: PLA/PBAT/TPS, PLA/PBAT/TPS-SA, and PLA/PBAT/TPS-MCSA. 

### 2.4. Characterization of Film

#### 2.4.1. Morphological Properties

The morphological characteristics of the film samples were meticulously investigated through the utilization of a Hitachi SU5000 scanning electron microscope. The film samples underwent preliminary embrittlement using liquid nitrogen. The unique attributes inherent to each sample were methodically observed under an applied accelerating voltage of 5.0 kV [20].

#### 2.4.2. Mechanical Properties

The mechanical properties of the films were meticulously assessed using an intelligent electronic tensile testing instrument (XLW (EC), Jinan, China) under controlled environmental conditions of 25 °C and 90% relative humidity (RH), following the ASTM- D882-12 (2012) [21]. To conduct the tests, the films were carefully cut into elongated strips measuring 150 mm × 15 mm. These samples were positioned between clamping points, initially set at a distance of 50 mm, and subjected to a controlled stretching rate of 50 mm/min.

#### 2.4.3. Barrier Properties

The oxygen transmission rate (OTR) of the film was determined concerning ASTM-D1434-82 (2015) [22]. The film was tailored to a uniform size using a special film cutter and placed in a sealed chamber. Three groups of films were tested 3 times at 23 °C and 50% RH, respectively. The water vapor transmission rate (WVTR) test for films was referred to ASTM-E398-13 (2013) [23]. Each group of films was examined three times at 38 °C and 10% RH.

#### 2.4.4. Optical Properties

The optical properties of the films were measured using an optical tester (WGT-S, Shanghai Precision Instrumentation Co., Shanghai, China) following the method of Hao et al. [24]. Different positions across the film were assessed for both transmittance (T) and haze (H). Each set of samples underwent five individual measurements, and the resulting average value was recorded.

### 2.5. Application to Banana Preservation

#### 2.5.1. Samples Preparation

Fresh bananas at a physiologically mature stage with a little green were procured from the Pudong Fresh Fruit and Vegetable Market in Shanghai on November 2nd. Bananas exhibiting consistent morphology and devoid of any physical damage were meticulously chosen for the evaluation of their freshness retention. The selected bananas were carefully inserted into prefabricated bags (size: 27 cm × 40 cm, thickness: 35 ± 3 μm) and hermetically sealed for storage purposes. The bananas were subsequently randomly allocated into four distinct groups, encompassing unwrapped bananas (designated as the control group) and bananas enveloped with PLA/PBAT/TPS, PLA/PBAT/TPS-SA, and PLA/PBAT/TPS-MCSA film. All samples were consistently stored at 25 ± 1 °C and 58% RH.

#### 2.5.2. Determination of CO_2_ and O_2_ Contents

The fractional content of O_2_/CO_2_ within the packaging was quantified using a CheckMate9900 headspace analyzer (Shanghai, China), according to the method of Pramod V. et al. [25]. The concentrations of O_2_ and CO_2_ within the packaging were gauged and documented by inserting a testing needle vertically into the package prior to its unsealing on each designated sampling day. The initial gases in the bag were all air, meaning that the oxygen was 21% and the carbon dioxide was nearly 0%.

#### 2.5.3. Rate of Weight Loss 

In order to assess the moisture retention capacity of the films on banana samples, the initial weight of each group of bananas was recorded prior to packaging. Over the course of 0 to 10 days of fruit storage, periodic weighing was conducted. The rate of weight loss (*RWL*) (%) was calculated using the following formula:

(1) $$RWL(\%)=\frac{M_{1}-M_{2}}{M_{1}}\times 100\%$$

where *M*_1_ is the mass of the banana before storage (g), and *M*_2_ is the mass of the banana after storage (g).

#### 2.5.4. Hardness

The hardness of the banana was assessed using a textural instrument (TA-XT-plus, Stable Micro Systems Co., Ltd., Surrey, UK). Three randomly chosen bananas from each group were measured at three distinct positions to ascertain their pulp firmness. Each group of samples underwent three measurements, and the resultant average value was calculated.

#### 2.5.5. Soluble Solids Content

The determination of soluble solids content (SSC) was carried out based on the methodology outlined by Fang et al. [26], with minor adaptations. Three bananas were randomly selected from each group; for every 2 d, a 20 g sample of banana was taken, ground into a slurry, and filtered through a filter cloth. The SSC content of the bananas was measured using a hand-held sugar refractometer (LH-T32, Lohand Biotech, Hangzhou, China).

#### 2.5.6. Malonic Dialdehyde

The malonic dialdehyde (MDA) content of the banana samples was measured following the method described by Li et al. [27]. Two grams of banana sample were placed into a mortar, followed by the addition of 12 mL of 100 g/L (*w*/*v*) trichloroacetic acid (TCA) for grinding into a slurry. After grinding into a slurry, the mixture was transferred to a centrifuge tube, and 6 mL of 6.7 g/L (*w*/*v*) thiobarbituric acid solution was added and thoroughly mixed. The resulting mixture was subjected to a 15 min reaction at 90 °C in a water bath, followed by rapid cooling and subsequent centrifugation at 12,000 rpm for 10 min. The absorbance of the supernatant was measured at wavelengths of 532 nm, 600 nm, and 450 nm.

#### 2.5.7. Polyphenol Oxidase

The detection of polyphenol oxidase (PPO) activity was slightly modified following the approach of Terefe et al. [28]. In a 0.1 M phosphate buffer (pH 6.8) containing 2% (*w*/*v*) poly (vinyl pyrrolidone), 5 mL of enzyme extraction solution was combined with 5 g of banana puree sample. After homogenization for 10 min, the mixture was subsequently centrifuged at 12,000 rpm for 30 min at 4 °C to isolate the supernatant. The absorbance of the supernatant at 420 nm was measured, and the results were recorded at 1 min intervals.

#### 2.5.8. Sensory Evaluation

The sensory evaluation of bananas was conducted following the methodology outlined by Corinna et al. [29]. For assessing the attributes of appearance, odor, and taste, a panel of six assessors (three males and three females) with specialized training in sensory evaluation was carefully chosen from the College of Food at Shanghai Ocean University. The training course consisted of two steps. In the first step, panelists were asked to fully familiarize themselves with the criteria for evaluating the appearance, odor, and taste of bananas. In the second step, all banana evaluations were conducted in an independent sensory evaluation lab and under a white light.

Sensory analyses were performed as independent replicates. Bananas were sampled at 2-day intervals and evaluated based on the sensory assessment criteria detailed in Table 2. A score below 60 indicated a loss of commercial value for the banana, while a score below 40 rendered it inedible.

### 2.6. Statistical Analyses

SPSS 26.0 software was used for one-way ANOVA, and the results were expressed as the mean ± standard deviation. *p* < 0.05 indicated significant differences, and Origin 2019 software was used for graphing.

## 3. Results and Discussion

### 3.1. Characterization of Film

#### 3.1.1. Microstructure Analysis

**Figure 1:**
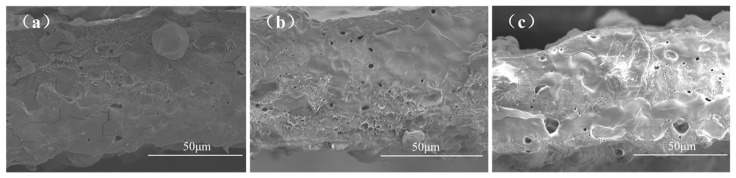
Microstructure of films: (**a**–**c**) PLA/PBAT/TPS, PLA/PBAT/TPS-SA, and PLA/PBAT/TPS-MCSA films.

Figure 1 illustrates the microstructure of the films. The cross-sections of the PLA/PBAT/TPS and PLA/PBAT/TPS-MCSA films exhibit rough and uneven features, while the cross-section of the PLA/PBAT/TPS-SA film appears smoother and more uniform than that of the PLA/PBAT/TPS and PLA/PBAT/TPS-MCSA films. This discrepancy arises from the reaction between SA and the TPS within the films. The carboxyl groups of SA react with the hydroxyl groups in the TPS structure, leading to a more orderly and organized molecular arrangement in the PLA/PBAT/TPS-SA film. In contrast, in the PLA/PBAT/TPS-MCSA films, the adsorption of SA onto MCM-41 reduces the contact area between SA and the film, resulting in a more disordered molecular structure. 

The microstructure of MCM-41 contains a substantial number of micropores, indirectly contributing to an increased level of surface roughness. Additionally, cross-sections of the PLA/PBAT/TPS-SA and PLA/PBAT/TPS-MCSA films feature unevenly distributed pores of varying sizes. This non-uniform distribution of pores can be attributed to the introduction of SA and MCSA powders, which lead to pore formation within the films. The uneven distribution of the powders in the film results in the formation of pores of varying sizes and non-uniform distribution.

#### 3.1.2. Mechanical Properties

The addition of the powder affected the overall tensile strength (TS) of the film (Table 3). With the addition of SA powder, the TS and elongation at break (EAB) of the PLA/PBAT/TPS-SA film showed a significant decrease; the longitudinal TS and transverse TS decreased by 30% and 46%, respectively, compared with the PLA/PBAT/TPS film; EAB decreased by 34% and 41%, respectively, compared with the PLA/PBAT/TPS-SA and PLA/PBAT/TPS films. The reason for this is that the carboxyl group in the SA molecule and the hydroxyl group in the TPS molecule undergo a condensation reaction, resulting in the denaturation of the TPS molecule, changes in the microstructure, and a decrease in mechanical strength. However, the mechanical properties of the PLA/PBAT/TPS-MCSA film were improved a little compared with the PLA/PBAT/TPS-SA film. This is because the adsorption of molecular sieve MCM-41 reduced the contact area of SA with starch, which reduced its damage to the starch structure and decreased the effect of starch denaturation on the mechanical properties of the film.

#### 3.1.3. Barrier Properties

Table 4 shows the OTR and WVTR of different films. The OTR of the PLA/PBAT/TPS-SA film was slightly decreased compared to the PLA/PBAT/TPS film. This was due to the polymerization of the hydroxyl group of TPS with the carboxyl group of SA, which reduced the pore space of the films. At the same time, a cross-linked network structure is formed, which is not conducive to oxygen penetration. As MCSA was added, the OTR of the PLA/PBAT/TPS-MCSA film increased to 543.1047 ± 3.468 cm^3^·m^−2^·24 h^−1^·0.1 MPa^−1^, relative to PLA/PBAT/TPS film (*p* < 0.05). This is accounted for by the adsorption of SA by MCM-41, which trapped SA in the pores, reducing its contact area with the films and preventing the formation of a tight cross-linked network. On the other hand, the pore structure of the molecular sieve is also more favorable for oxygen penetration, which is consistent with the SEM results.

Foodstuffs are prone to water loss when exposed, leading to drying and dehydration denaturation, and WVTR is one of the indicators that can effectively evaluate the barrier performance of films. The WVTR of the PLA/PBAT/TPS-SA film increased by 37% compared to the PLA/PBAT/TPS film, while the PLA/PBAT/TPS-MCSA film decreased by 20% compared to PLA/PBAT/TPS-SA. The reason is that the irregular microstructure of SA led to the increase in pores between the film substrates; meanwhile, the benzene ring in SA, as a typical hydrophobic group, reduced the affinity between starch and water molecules, which reduced the hygroscopicity of starch and allowed water vapor to escape from the film freely. However, the microporous structure of MCM-41 adsorbed a certain amount of water, which could effectively trap part of the volatile water vapor, maintain the balance of microenvironment moisture, and reduce the water loss and denaturation of food. Therefore, the PLA/PBAT/TPS-MCSA films are more suitable for the preservation of fruits and vegetables compared to the PLA/PBAT/TPS-SA films.

#### 3.1.4. Optical Properties

The T and H of the film are important factors that affect the aesthetics of the film and the experience of buying it for consumers [30]. Table 5 shows the optical properties of the three groups of films. Compared with the PLA/PBAT/TPS film, the T of the PLA/PBAT/TPS-SA film was slightly lower, but there was no significant difference; the T of the PLA/PBAT/TPS-MCSA film decreased by 23.3% (*p* < 0.05). This is in agreement with the results of Mohit J. et al. [31] in the study of the addition of salicylic acid (SA) to UV, antioxidant, and antimicrobial gelatin films. With the addition of salicylic acid and its increasing content, the transmittance of gelatin films gradually decreased, and UV-blocking ability gradually increased. The SA crystal surface was smoother, while the surface of MCM-41 was rougher and aggregated. The pore structure of MCM-41 provided a larger specific surface area and an irregular microstructure, which increased the refractive index of light and decreased the T. Similarly, compared to the PLA/PBAT/TPS film, the H of the PLA/PBAT/TPS-MCSA film increased by about 20% (*p* < 0.05), indicating a better absorption of visible light in the PLA/PBAT/TPS-MCSA film.

### 3.2. Qualities of Bananas

#### 3.2.1. Volume Fractions of O_2_ and CO_2_ Contents

**Figure 2:**
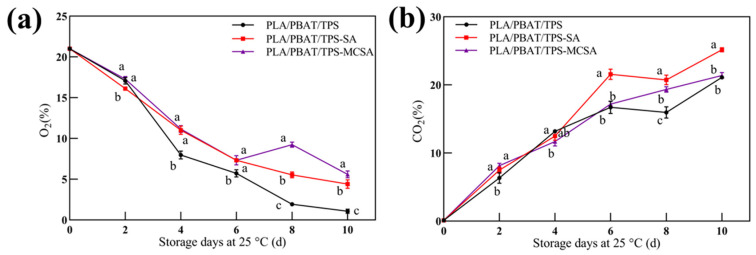
Gas composition: (**a**) O_2_ volume fraction; (**b**) CO_2_ volume fraction. Different letters indicate significant differences within each parameter (*p* < 0.05).

Within a specified range, an increase in film OTR can regulate the oxygen-to-carbon dioxide ratio within the packaging [32], thereby facilitating the preservation of bananas. As shown in Figure 2, the concentration of O_2_ in the packages decreased continuously throughout the storage period, while the concentration of CO_2_ increased continuously. The volumetric fraction of oxygen within the bag exhibits significant fluctuations during the 6th to 10th day, corresponding to the accelerated metabolic and enhanced respiratory activities of the bananas. During this preservation phase, the PLA/PBAT/TPS film created a microenvironment characterized by reduced oxygen and elevated carbon dioxide levels, resulting in the overall respiration rate of the banana being maintained at a low level [33]. Valeria et al. [34] prepared a zeolite-loaded salicylic acid that could be uniformly distributed in the bio-based plastic polyamide 11 (PA11) and ensured the activity and slow release of SA. However, the active packaging film reduced gas permeability, which was detrimental to the preservation of respiratory leap fruits and vegetables, such as bananas. In contrast to the PLA/PBAT/TPS-SA film, the PLA/PBAT/TPS-MCSA film possessed heightened oxygen permeability, enabling gas exchange with the environment. This intrinsic capacity allows the PLA/PBAT/TPS-MCSA film to spontaneously regulate the gas composition within the film, slowing down the aging and deterioration of bananas in the packaging film to achieve an effect of preservation and consistency with the design principles of packaging films applied to fruit and vegetable storage and preservation. 

#### 3.2.2. Rate of Weight Loss 

**Figure 3:**
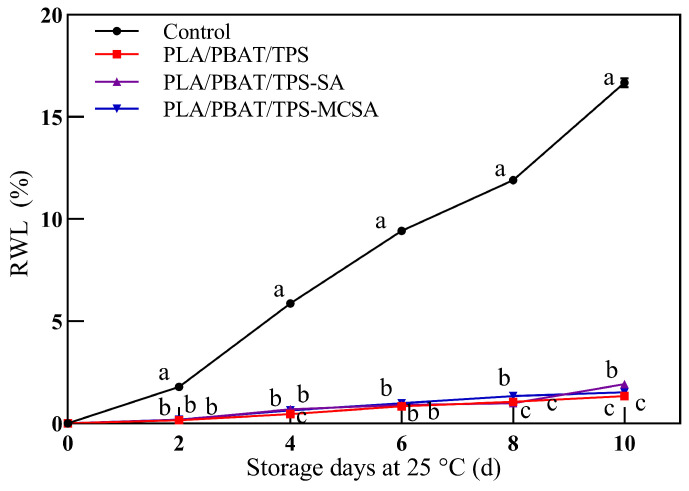
Effect of different packages on RWL of bananas. Different letters indicate significant differences within each parameter (*p* < 0.05).

During the storage period, transpiration and respiration processes in fruits can result in moisture loss, leading to a decline in quality. As illustrated in Figure 3, over the course of storage, all three film groups exhibited superior capability, compared to the control group, in maintaining the epidermal strength of bananas. This effectiveness in preserving epidermal integrity aids in mitigating moisture loss, consequently reducing overall fruit quality degradation. Remarkably, there are no significant differences observed among the three film groups with regard to the RWL in the preserved bananas (*p* < 0.05). Zhang et al. [35] significantly delayed the reduction of RWL in post-harvest strawberries using a combined treatment of blue light and salicylic acid (BL + SA), which successfully demonstrated the importance of SA for fruit preservation.

#### 3.2.3. Hardness

**Figure 4:**
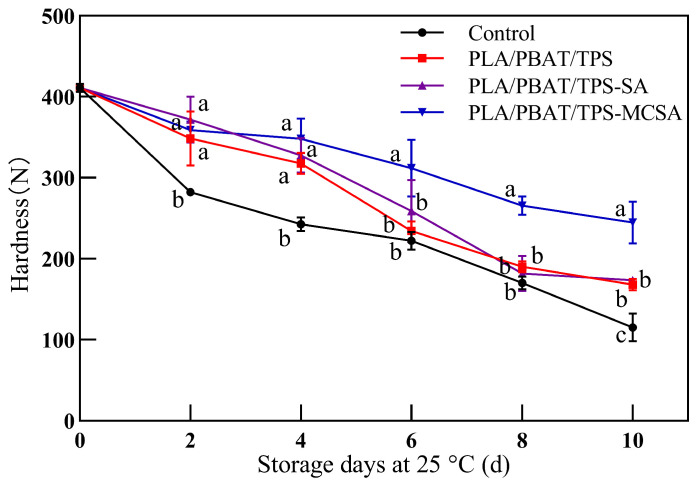
Effect of different packages on hardness of bananas. Different letters indicate significant differences within each parameter (*p* < 0.05).

Throughout the storage process, bananas experienced a decline in pulp hardness due to the depletion of nutrients, accompanied by the generation of moisture and carbon dioxide, leading to the onset of spoilage and softening. Thus, hardness serves as a valuable indicator for evaluating banana storage quality [2]. As depicted in Figure 4, a conspicuous reduction in banana pulp hardness is evident with increasing storage duration. Throughout the entire storage period, the hardness of the control group bananas consistently remained lower than that of the other groups. This phenomenon is attributed to the protective influence of the packaging films, which notably attenuates the rate of banana softening. The decrease in hardness indicates the softening of the fruit, which is associated with dehydration and breaking of the cellular structure [36]. Notably, in the case of the PLA/PBAT/TPS-MCSA film, starting from the 6th day of storage, the banana pulp hardness surpassed that of the PLA/PBAT/TPS-SA and PLA/PBAT/TPS-MCSA films, exhibiting significant differences (*p* < 0.05). This distinctive trend arises from the high oxygen permeability of the PLA/PBAT/TPS-MCSA film, conferring it with superior gas modulation capabilities. Additionally, the enhanced respiration of bananas expedites the dissolution of MCSA powder within the PLA/PBAT/TPS-MCSA film. Consequently, the embedded SA molecules are effectively released, retarding the oxidative consumption of nutritional substances within the bananas. Consequently, this mechanism contributes to the deceleration of banana softening.

#### 3.2.4. Soluble Solids Content

**Figure 5:**
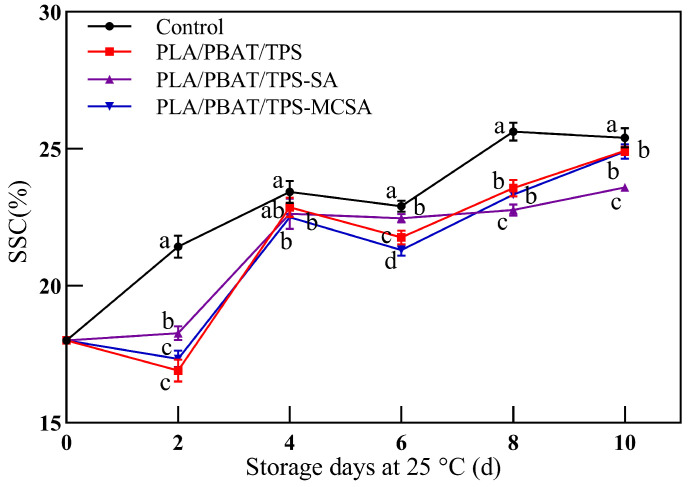
Effect of different packages on SSC of bananas. Different letters indicate significant differences within each parameter (*p* < 0.05).

As shown in Figure 5, the SSC of bananas displays an upward trend throughout the storage period. SSC is one of the signs of a high respiration rate and is related to the aging process of fruits and vegetables [37,38]. The oxidative breakdown of nutrients in bananas, such as polysaccharides and lipids, to produce soluble polysaccharides and lipids, as well as the flow of banana contents by cell wall degrading enzymes, have led to an increase in banana SSC [39]. In addition, the solubilization of cell wall polysaccharides and hemicellulose could increase the soluble solids content of bananas [39]. The results of the SSC demonstrate that the PLA/PBAT/TPS-SA and PLA/PBAT/TPS-MCSA films exhibit a more favorable effect in retarding the ripening and deterioration of bananas during storage compared to the PLA/PBAT/TPS film. This result aligns with the observations of banana hardness, as illustrated in Figure 4.

#### 3.2.5. Malonic Dialdehyde

**Figure 6:**
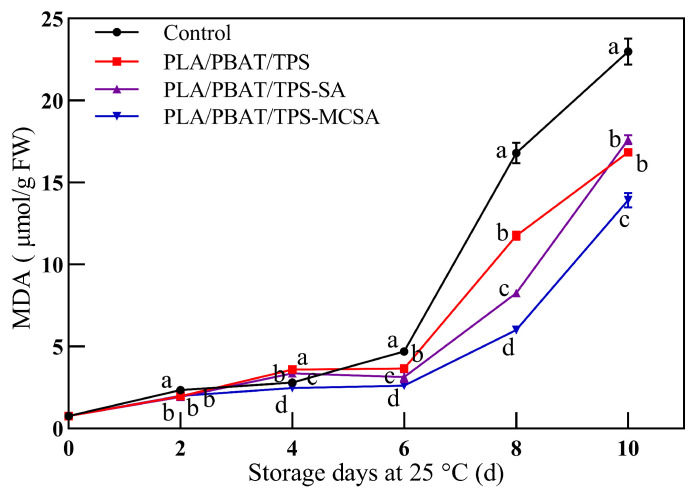
Effect of different packages on the content of MDA in bananas. Different letters indicate significant differences within each parameter (*p* < 0.05).

The level of MDA content can reflect the integrity of banana cell membranes and, indirectly, the quality of the banana [40]. During storage, the MDA content of all banana samples gradually increased, as revealed in Figure 6, indicating a progressive peroxidation of membrane lipids. From the 6th day onwards, the MDA content of bananas packaged in the PLA/PBAT/TPS-MCSA film was significantly lower than that of the other groups (*p* < 0.05). This could be attributed to the interaction between bananas and the active component SA, which is released from the PLA/PBAT/TPS-MCSA film during the preservation period, suppressing the generation of reactive oxygen species (ROS). Consequently, the levels of MDA, a principal product of ROS, decrease correspondingly, thereby reducing its impact on banana cell membranes [41]. In the late preservation period, the ability of the PLA/PBAT/TPS-SA film to delay banana peroxidation decreased, which is attributed to the lower oxygen concentration within the films (Figure 2), which is more conducive to restraining the accumulation of MDA. The results demonstrate that the prepared PLA/PBAT/TPS-MCSA film effectively safeguards the cell membrane system and maintains the quality of the banana.

#### 3.2.6. Polyphenol Oxidase 

**Figure 7:**
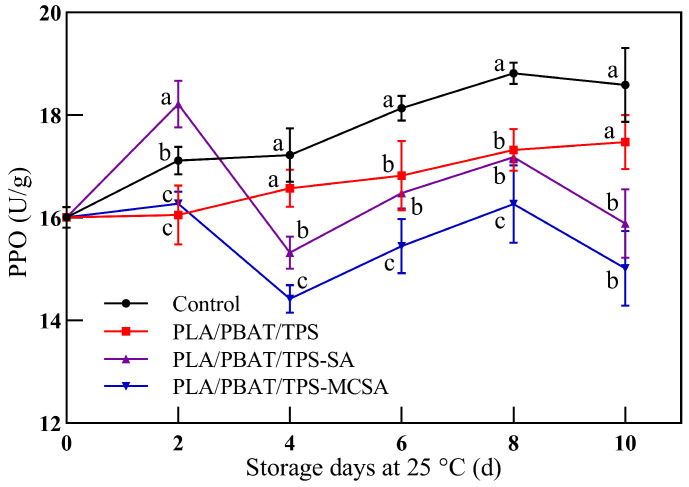
Effect of different packages on PPO activity of bananas. Different letters indicate significant differences within each parameter (*p* < 0.05).

PPO, as the primary cause of browning in fruits and vegetables, plays a crucial role in the process of polyphenol oxidation [42,43]. PPO catalyzes the oxidation of polyphenols to quinones, which then polymerize and react with intracellular amino acids to form melanin, leading to browning [44]. The direct impact of highly active PPO on bananas is evident in the degree of browning observed in both the skin and pulp. Throughout the entire storage period, the PLA/PBAT/TPS-MCSA film demonstrated the most effective inhibition of PPO activity. As depicted in Figure 7, the initial rise in PPO activity in bananas wrapped in the PLA/PBAT/TPS-MCSA film may be associated with the sudden release of accumulated PPO from vacuoles [45]. However, the PPO content in bananas enclosed by the PLA/PBAT/TPS-MCSA film remains consistently lower than in the other groups, highlighting the film’s capability to preserve the integrity of cell membranes. This observation also indicates that the PLA/PBAT/TPS-MCSA film can act as a membrane barrier between polyphenol oxidase and phenolic substances, thus reducing enzyme activity and minimizing phenolic oxidation. 

#### 3.2.7. Sensory Evaluation

**Figure 8:**
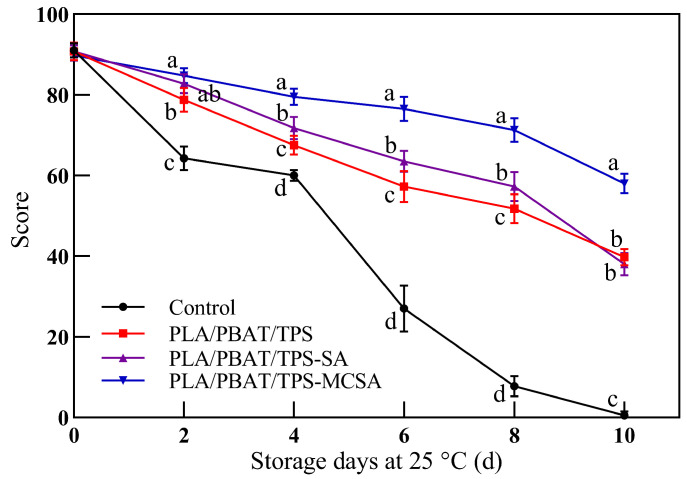
Sensory evaluation of bananas in the naked group and in different bags. Different letters indicate significant differences within each parameter (*p* < 0.05).

Sensory evaluation of bananas refers to the comprehensive assessment of fruit based on human sensory organs, including olfaction, tactile sensations, and visual perceptions, making it a direct indicator of their commercial value [46]. As depicted in Figure 8, the overall storage quality of bananas gradually declines over the storage period, accompanied by a decrease in sensory scores. Among the groups, the control bananas exhibit the fastest rate of sensory score decline, becoming commercially valueless by the fourth day of storage and inedible by the sixth day. Bananas from the PLA/PBAT/TPS and PLA/PBAT/TPS-SA films maintained their edibility until the tenth day, whereas those from the PLA/PBAT/TPS-MCSA film retained favorable sensory scores even towards the end of storage, preserving both commercial and edible value. The positive and proactive impact of the PLA/PBAT/TPS-MCSA film on banana preservation is evident. It significantly extends the storage period of bananas, thereby reducing commercial losses caused by spoilage. The results indicate that the PLA/PBAT/TPS-MCSA film can effectively prolong the shelf life of bananas by 4–5 days.

## 4. Conclusions

In this study, a biodegradable, gas-regulating packaging film was developed by incorporating the natural plant growth regulator, SA, into MCM-41, and its application was extended to the preservation of bananas. The results reveal that in comparison to the PLA/PBAT/TPS films, the OTR of the PLA/PBAT/TPS-MCSA films increased from 384.36 ± 22.06 cm^3^·m^−2^·24 h^−1^·0.1 MPa^−1^ to 543.10 ± 3.47 cm^3^·m^−2^·24 h^−1^·0.1 MPa^−1^. At 25 ± 1 °C, the PLA/PBAT/TPS-MCSA film demonstrated favorable preservation effects on banana quality, effectively delaying the increase in RWL, SSC, MDA, and PPO levels. Consequently, the shelf life of bananas could be extended by 4–5 days. The innovative, biodegradable packaging developed in this research provides a promising approach for future gas-regulated packaging of fruits and vegetables. To elucidate the regulatory role of MCSA in banana preservation, further investigations could be conducted to assess the impact of MCSA on the endogenous enzyme activities within bananas.

## Figures and Tables

**Table 1:** Formulation of composite films.

Sample	PLA (wt%)	PBAT (wt%)	TPS (wt%)	SA (wt%)	MCSA (wt%)
PLA/PBAT/TPS	15	45	40	0	0
PLA/PBAT/TPS-SA	14.7	44.1	39.2	2	0
PLA/PBAT/TPS-MCSA	14.7	44.1	39.2	0	2

Where PLA, PBAT, TPS, SA, and MCSA indicate poly (lactic acid) (PLA), poly (butylene adipate-co-terephthalate) (PBAT), thermoplastic starch (TPS), salicylic acid (SA), and mobil composition of matter (MCM)-41 loaded with salicylic acid (MCSA), respectively.

**Table 2:** The sensory evaluation of bananas.

Score	Basis of Scoring
81~100	Good taste of flesh, bright yellow peel color, no mechanical damage, no angles, no brown.
61~80	The peel is dark yellow, with tiny needle-like brown spots, the flesh has a good taste and no mechanical damage.
41~60	Sesame-sized brown spots on the peel, soft flesh texture.
21~40	Large, localized brown spots on the epidermis, tawny, slight mechanical damage to the flesh.
1~20	Large browning on the surface, soft flesh, and unpleasant odor.
0	Large browning of the epidermis, severe flesh decay, irritating odor, inedible.

**Table 3:** Mechanical properties of films.

Sample	TS (Vertical) (MPa)	EAB (Vertical) (%)	TS (Lateral) (MPa)	EAB (Lateral) (%)
PLA/PBAT/TPS	30.28 ± 0.958 ^c^	436.56 ± 23.780 ^c^	26.11 ± 2.732 ^c^	356.44 ± 16.670 ^c^
PLA/PBAT/TPS-SA	21.20 ± 0.549 ^a^	288.79 ± 29.550 ^a^	12.12 ± 2.025 ^a^	211.34 ± 13.770 ^a^
PLA/PBAT/TPS-MCSA	26.93 ± 0.828 ^b^	379.56 ± 35.460 ^b^	16.86 ± 1.421 ^b^	256.79 ± 23.670 ^b^

Different superscript letters (a–c) between the same columns indicate significant differences (*p* < 0.05).

**Table 4:** Barrier properties of films.

Sample	OTR (cm^3^·m^−2^·24 h^−1^·0.1 MPa^−1^)	WVTP (g·m^−1^·Pa^−1^·s^−1^)
PLA/PBAT/TPS	384.36 ± 22.06 ^b^	860.08 ± 4.11 ^a^
PLA/PBAT/TPS-SA	361.29 ± 3.12 ^a^	1178.13 ± 37.67 ^c^
PLA/PBAT/TPS-MCSA	543.10 ± 3.47 ^c^	938.50 ± 12.38 ^b^

Different superscript letters (a–c) between the same columns indicate significant differences (*p* < 0.05).

**Table 5:** Optical properties of films.

Sample	T (%)	H (%)
PLA/PBAT/TPS	30.61 ± 0.81 ^b^	43.58 ± 0.74 ^a^
PLA/PBAT/TPS-SA	29.90 ± 0.70 ^b^	42.15 ± 0.93 ^a^
PLA/PBAT/TPS-MCSA	23.37 ± 1.07 ^a^	53.20 ± 0.39 ^b^

Different superscript letters (a,b) between the same columns indicate significant differences (*p* < 0.05).

## Data Availability

Data is contained within the article.
